# AlphaFold Protein Structure Database 2025: a redesigned interface and updated structural coverage

**DOI:** 10.1093/nar/gkaf1226

**Published:** 2025-11-22

**Authors:** Damian Bertoni, Maxim Tsenkov, Paulyna Magana, Sreenath Nair, Ivanna Pidruchna, Marcelo Querino Lima Afonso, Adam Midlik, Urmila Paramval, Dare Lawal, Ahsan Tanweer, Meera Last, Risha Patel, Agata Laydon, Dariusz Lasecki, Nick Dietrich, Hamish Tomlinson, Augustin Žídek, Tim Green, Oleg Kovalevskiy, Andy Lau, Shaun Kandathil, Nicola Bordin, Ian Sillitoe, Milot Mirdita, David Jones, Christine Orengo, Martin Steinegger, Jennifer R Fleming, Sameer Velankar

**Affiliations:** European Molecular Biology Laboratory, European Bioinformatics Institute, Hinxton, CB10 1SD,United Kingdom; European Molecular Biology Laboratory, European Bioinformatics Institute, Hinxton, CB10 1SD,United Kingdom; European Molecular Biology Laboratory, European Bioinformatics Institute, Hinxton, CB10 1SD,United Kingdom; European Molecular Biology Laboratory, European Bioinformatics Institute, Hinxton, CB10 1SD,United Kingdom; European Molecular Biology Laboratory, European Bioinformatics Institute, Hinxton, CB10 1SD,United Kingdom; European Molecular Biology Laboratory, European Bioinformatics Institute, Hinxton, CB10 1SD,United Kingdom; European Molecular Biology Laboratory, European Bioinformatics Institute, Hinxton, CB10 1SD,United Kingdom; European Molecular Biology Laboratory, European Bioinformatics Institute, Hinxton, CB10 1SD,United Kingdom; European Molecular Biology Laboratory, European Bioinformatics Institute, Hinxton, CB10 1SD,United Kingdom; European Molecular Biology Laboratory, European Bioinformatics Institute, Hinxton, CB10 1SD,United Kingdom; Google DeepMind, London, N1C 4DN,United Kingdom; Google DeepMind, London, N1C 4DN,United Kingdom; Google DeepMind, London, N1C 4DN,United Kingdom; Google DeepMind, London, N1C 4DN,United Kingdom; Google DeepMind, London, N1C 4DN,United Kingdom; Google DeepMind, London, N1C 4DN,United Kingdom; Google DeepMind, London, N1C 4DN,United Kingdom; Google DeepMind, London, N1C 4DN,United Kingdom; Google DeepMind, London, N1C 4DN,United Kingdom; Department of Computer Science, University College London, London, WC1E 6BT,United Kingdom; Department of Computer Science, University College London, London, WC1E 6BT,United Kingdom; Institute of Structural and Molecular Biology, University College London, London, England, WC1E 6BT,United Kingdom; Institute of Structural and Molecular Biology, University College London, London, England, WC1E 6BT,United Kingdom; School of Biological Sciences, Seoul National University, Seoul08826, Republic of Korea; Department of Computer Science, University College London, London, WC1E 6BT,United Kingdom; Institute of Structural and Molecular Biology, University College London, London, England, WC1E 6BT,United Kingdom; School of Biological Sciences, Seoul National University, Seoul08826, Republic of Korea; Interdisciplinary Program in Bioinformatics, Seoul National University, Seoul08826, Republic of Korea; Institute of Molecular Biology and Genetics, Seoul08826, Republic of Korea; Artificial Intelligence Institute, Seoul National University, Seoul08826, Republic of Korea; European Molecular Biology Laboratory, European Bioinformatics Institute, Hinxton, CB10 1SD,United Kingdom; European Molecular Biology Laboratory, European Bioinformatics Institute, Hinxton, CB10 1SD,United Kingdom

## Abstract

The AlphaFold Protein Structure Database (AFDB; https://alphafold.ebi.ac.uk), developed by EMBL–EBI and Google DeepMind, provides open access to hundreds of millions of high-accuracy protein structure predictions, transforming research in structural biology and the wider life sciences. Since its launch, AFDB has become a widely used bioinformatics resource, integrated into major databases, visualization platforms, and analysis pipelines. Here, we report the update of the database to align with the UniProt 2025_03 release, along with a comprehensive redesign of the entry page to enhance usability, accessibility, and structural interpretation. The new design integrates annotations directly with an interactive 3D viewer and introduces dedicated domains and summary tabs. Structural coverage has also been updated to include isoforms plus underlying multiple sequence alignments. Data are available through the website, FTP, Google Cloud, and updated APIs. Together, these advances reinforce AFDB as a sustainable resource for exploring protein sequence–structure relationships.

## Graphical Abstract

**Graphical Abstract:**
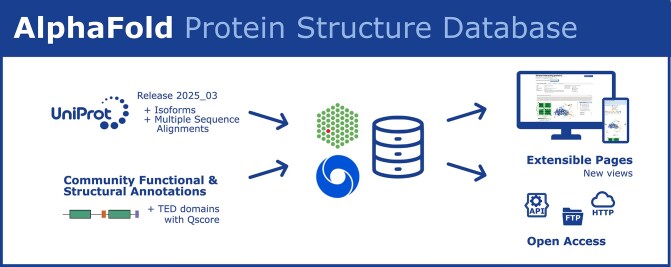

## Introduction

The field of life science has been transformed by protein structure prediction tools, such as AlphaFold [1–3], ESMFold [4], and RoseTTAFold [5]. Built upon decades of foundational research [6–8] and open scientific data resources such as the Protein Data Bank (PDB) [9, 10], MGnify [11] and the Universal Protein Resource (UniProt) [12], these advanced methods have dramatically narrowed the gap between the vast number of known protein sequences and the availability of structure models.

The AlphaFold Protein Structure Database (AFDB), a collaboration between Google DeepMind and EMBL–EBI [13–15], was established to democratize access to millions of highly accurate protein structure predictions. The practical utility of these predictions is underscored by their use, with to date, over 4,501,953 total users accessing the AFDB website, more than 18,000 proteome archives downloaded, and the data's integration into major primary data resources [16, 17] and molecular visualization software [18–20]. This widespread adoption has benefited various life science disciplines from structural biology and bioinformatics to drug discovery [21–27].

To meet the ongoing demand for comprehensive structural data, AFDB has continued to evolve with new tools and functionalities [15]. However, synchronization with UniProt fell several years behind, leaving the database increasingly out of date and limiting coverage of newly described proteins [28]. The current release restores alignment with UniProt (2025_03), ensuring that predictions once again reflect the evolving sequence space. Newly generated multiple sequence alignments (MSAs) are provided, and coverage has been extended to include isoform-specific predictions, broadening biological relevance and accuracy. In parallel, a redesigned entry page enhances usability and interpretation.

## Enhanced user experience and functionalities

**Figure 1.**
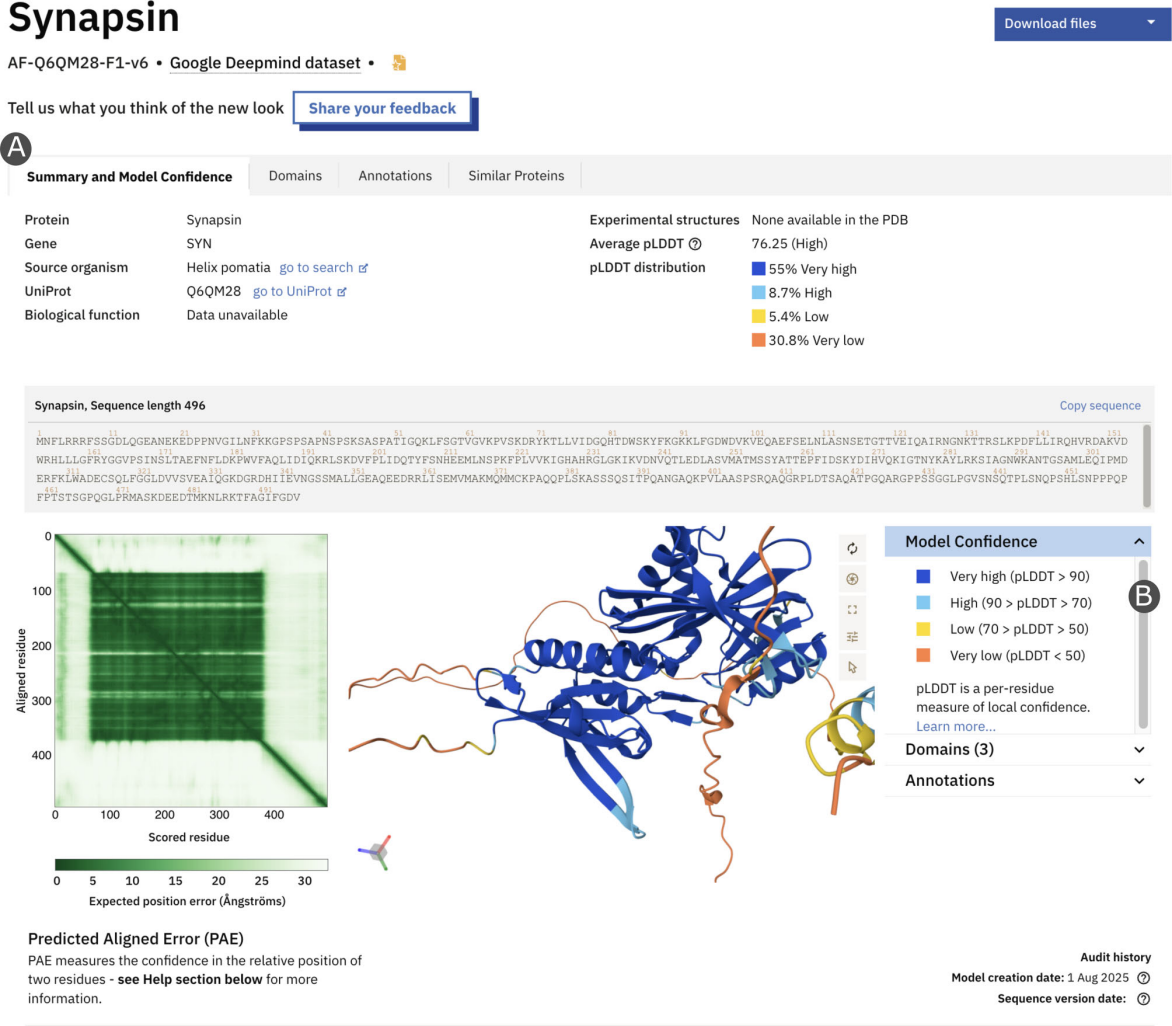
Example of summary page for Synapsin AF-Q6QM28-F1-v6. (**A**) The page is now arranged with tabs separating each section. The central panel displays the predicted 3D model, coloured by per-residue confidence score (pLDDT). (**B**) The right panel accordion provides an overview of domains and annotations and allows the user to toggle between different colour schemes.

To prioritize an intuitive and accessible user experience, AFDB has now undergone a redesign (Fig. 1). A central element of this overhaul is the enhanced prominence of the structure viewer, now featuring integrated annotations for a more streamlined analysis. New, focused tabs, such as the new Domains tab, are directly linked to the 3D viewer, simplifying complex visualization and analysis by providing immediate structural insights. This functionality, developed with accessibility and usability principles in mind, lowers the entry barrier for expert and non-expert users, making use of advanced structural biology data more inclusive and impactful for the global scientific community. This commitment to user-centred design ensures that the powerful insights from AlphaFold predictions are readily available and comprehensible to a broad audience.

## Data update

The AFDB collection has now been updated to UniProt release (2025_03), incorporating Swiss-Prot/UniProtKB isoforms. The integration of isoforms offers a more complete view of proteome diversity and has revealed widespread changes in protein domain architecture and interaction interfaces [29, 30]. Furthermore, we now provide the underlying MSAs and MSA depths used to predict the structures, offering a new layer of data for evolutionary and structural analyses.

## Integrating the Encyclopedia of Domains

**Figure 2.**
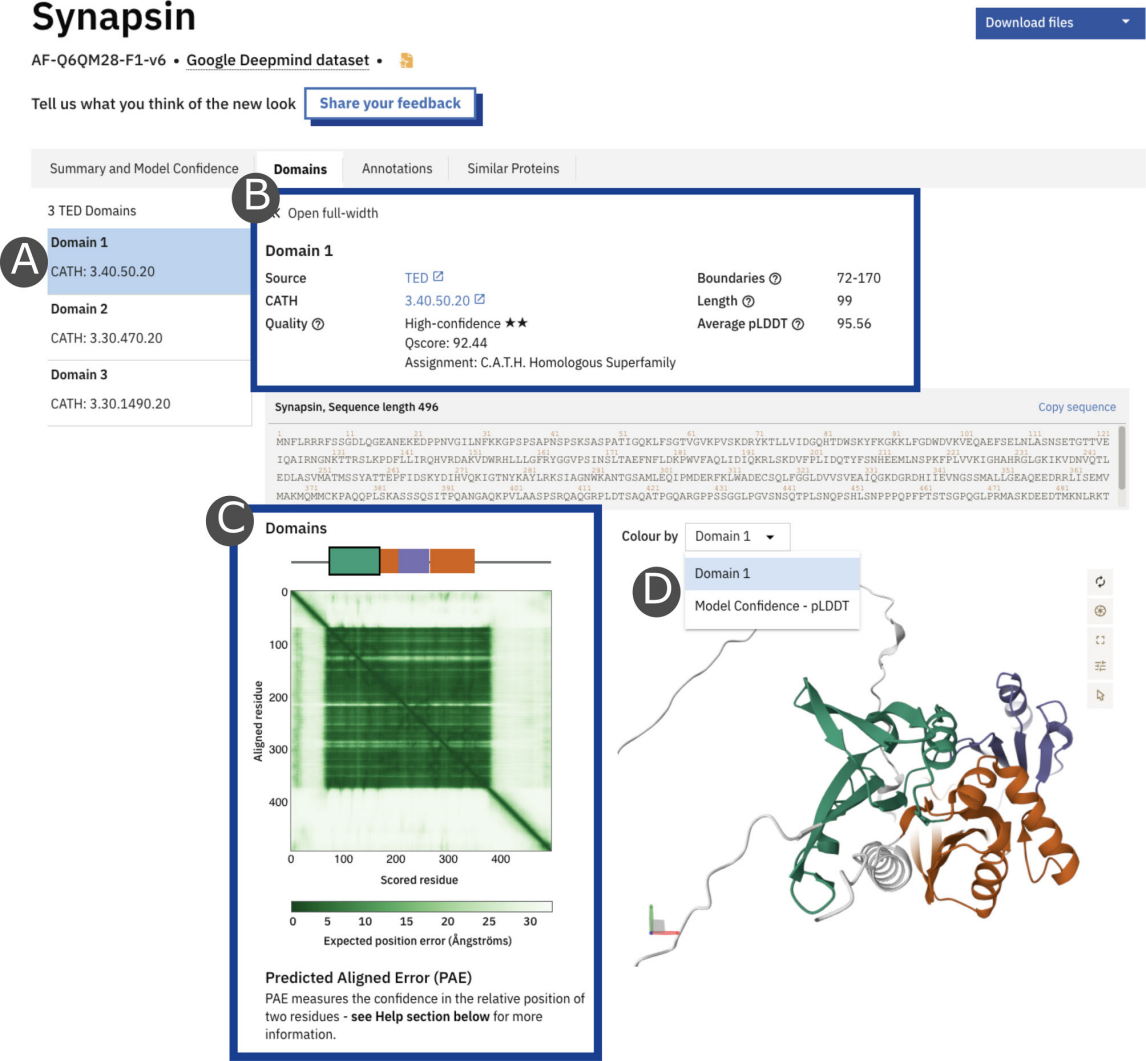
Example of domains tab for Synapsin AF-Q6QM28-F1-v6. This tab provides a comprehensive overview of the information available for a single protein domain provided by TED in the AFDB. (**A**) The panel on the left shows the complete list of domains identified in the entry. (**B**) The descriptive and quality assessment key metrics for a specific domain, including boundaries, length, mean pLDDT, and Qscore, are shown along with the high-confidence CATH assignment. (**C**) The predicted aligned error plot provides a visual representation of the AF2-predicted confidence in the orientation of the domains relative to the rest of the protein. (**D**) Colours on the model can be toggled between domain and pLDDT.

To facilitate functional understanding of the proteins in AFDB, over 361 million domain annotations from the Encyclopedia of Domains (TED) [21] covering over 165M proteins have now been added. The TED methodology employs a state-of-the-art, consensus-based approach that integrates three deep-learning-based domain parsing algorithms to robustly determine domain boundaries. These domain assignments are subsequently aligned against the CATH database (Version 4.4) [31], using structural alignment algorithms. Each domain entry in the AFDB is associated with a suite of descriptive and quality assessment metrics (Fig. 2). A consensus level serves as an initial quality indicator, reflecting the degree of agreement among the three domain-parsing methods. For domains with a structural match, a CATH identifier and assignment are provided based on the level of the hierarchy matched. A second key metric, the Qscore, provides a comprehensive, composite measure of the quality of each domain assignment (details in Supplementary data). This resource supports evolutionary, structural, and functional studies by providing a deeper view of domain organization.

## Data availability and API updates

The AFDB prioritizes open data access. All data, including new additions, are accessible via our website (https://alphafold.ebi.ac.uk). The EMBL–EBI’s FTP area hosts TAR files for proteomes of 46 organisms, including model organisms and WHO pathogens of interest (ftp.ebi.ac.uk/pub/databases/alphafold). The full AlphaFold dataset is accessible from Google Cloud Public Datasets, with user guidance at https://alphafold.ebi.ac.uk/download and version history documented at https://ftp.ebi.ac.uk/pub/databases/alphafold/CHANGELOG.txt. To support continued development, AFDB is introducing breaking changes to its API schema. The legacy API will be retired in June 2026 (https://alphafold.ebi.ac.uk/api-docs).

## Discussion and future directions

The latest updates to AFDB bring the resource back in line with UniProt and introduce an extensible interface designed to support future developments that enrich data and functionality. The inclusion of isoform-specific predictions and newly generated MSAs expands structural coverage and adds essential biological and evolutionary context to the models. This, in turn, lowers the barrier to entry for structure-informed research across the life sciences, enhances reproducibility, and promotes sustainable and greener research practices.

Looking ahead, future releases will continue to work with the scientific community to focus on new datasets and annotations, ensuring that AFDB reflects both the expanding sequence space and the evolving needs of researchers. Its development is guided by three principles: (i) filling gaps in structural coverage, (ii) improving existing models to enhance accuracy and utility, and (iii) working with the scientific community to address global challenges, from antimicrobial resistance to food security. A key priority is the inclusion of structural models and their validation in ways that maximize their scientific impact. Central to this vision is fostering community participation in annotation, building on the successful PDBe-KB model of collaborative consortia. By coupling structural predictions with rich, community-contributed functional insights, AFDB will continue to evolve into a knowledge-rich resource that accelerates discovery and amplifies the impact of protein structure data.

## Supplementary Material

gkaf1226_Supplemental_File

## Data Availability

The AFDB is available at https://alphafold.ebi.ac.uk.
