# Supplementary material for: AlphaFold Protein Structure Database 2025: a redesigned interface and updated structural coverage
Source: Nucleic Acids Res. 2025 Nov 22;54(D1):D358–62. doi: 10.1093/nar/gkaf1226 (PMC12807749; doi:10.1093/nar/gkaf1226)
Supplement: gkaf1226_Supplemental_File [file gkaf1226_supplemental_file.docx]

**AlphaFold DB v6 release and UniProt alignment**

AlphaFold DB version 6 (released October 2025) restores synchronisation with UniProt release 2025_03. This update increased the total number of predicted structures from 214,683,829 (v4) to 241,070,489 (v6). Relative to v4, 65,711,653 entries were added (including new proteins and isoforms), 39,324,993 entries were removed following UniProt archival, 276,539 sequences were modified, and 175,082,297 entries were carried over without sequence change but updated to reflect the latest UniProt metadata.

Only sequences that were new or changed between UniProt versions were refolded; models for unchanged sequences were retained from v4. Therefore, the alignment with UniProt did not retrospectively improve existing predictions (e.g., those with low MSA depth), but rather expanded the database to include predictions for newly released or updated sequences, ensuring the resource continues to reflect the evolving sequence space.

Further details, including accession lists for added, removed, changed, and unchanged entries, are available from the AlphaFold DB FTP site (<https://ftp.ebi.ac.uk/pub/databases/alphafold/>).

**Supplementary Table 1 - Proteomes available for download in bulk from FTP**

| **Species** | **Common Name** | **Reference Proteome** | **Predicted Structures** | **Download** |
| --- | --- | --- | --- | --- |
| *Ajellomyces capsulatus* | Ajellomyces capsulatus | [UP000001631](https://www.uniprot.org/proteomes/UP000001631) | 9,199 | [1,363 MB](https://ftp.ebi.ac.uk/pub/databases/alphafold/latest/UP000001631_447093_AJECG_v6.tar) |
| *Brugia malayi* | Brugia malayi | [UP000006672](https://www.uniprot.org/proteomes/UP000006672) | 10,972 | [1,635 MB](https://ftp.ebi.ac.uk/pub/databases/alphafold/latest/UP000006672_6279_BRUMA_v6.tar) |
| *Campylobacter jejuni* | *C. jejuni* | [UP000000799](https://www.uniprot.org/proteomes/UP000000799) | 1,620 | [175 MB](https://ftp.ebi.ac.uk/pub/databases/alphafold/latest/UP000000799_192222_CAMJE_v6.tar) |
| *Cladophialophora carrionii* | Cladophialophora carrionii | [UP000094526](https://www.uniprot.org/proteomes/UP000094526) | 11,170 | [1,729 MB](https://ftp.ebi.ac.uk/pub/databases/alphafold/latest/UP000094526_86049_9EURO1_v6.tar) |
| *Dracunculus medinensis* | Dracunculus medinensis | [UP000274756](https://www.uniprot.org/proteomes/UP000274756) | 10,834 | [1,364 MB](https://ftp.ebi.ac.uk/pub/databases/alphafold/latest/UP000274756_318479_DRAME_v6.tar) |
| *Fonsecaea pedrosoi* | Fonsecaea pedrosoi | [UP000053029](https://www.uniprot.org/proteomes/UP000053029) | 12,509 | [2,014 MB](https://ftp.ebi.ac.uk/pub/databases/alphafold/latest/UP000053029_1442368_9EURO2_v6.tar) |
| *Haemophilus influenzae* | *H. influenzae* | [UP000000579](https://www.uniprot.org/proteomes/UP000000579) | 1,660 | [175 MB](https://ftp.ebi.ac.uk/pub/databases/alphafold/latest/UP000000579_71421_HAEIN_v6.tar) |
| *Helicobacter pylori* | *H. pylori* | [UP000000429](https://www.uniprot.org/proteomes/UP000000429) | 1,540 | [166 MB](https://ftp.ebi.ac.uk/pub/databases/alphafold/latest/UP000000429_85962_HELPY_v6.tar) |
| *Klebsiella pneumoniae* | *K. pneumoniae* | [UP000007841](https://www.uniprot.org/proteomes/UP000007841) | 5,727 | [559 MB](https://ftp.ebi.ac.uk/pub/databases/alphafold/latest/UP000007841_1125630_KLEPH_v6.tar) |
| *Leishmania infantum* | *L. infantum* | [UP000008153](https://www.uniprot.org/proteomes/UP000008153) | 7,924 | [1,508 MB](https://ftp.ebi.ac.uk/pub/databases/alphafold/latest/UP000008153_5671_LEIIN_v6.tar) |
| *Madurella mycetomatis* | Madurella mycetomatis | [UP000078237](https://www.uniprot.org/proteomes/UP000078237) | 9,561 | [1,537 MB](https://ftp.ebi.ac.uk/pub/databases/alphafold/latest/UP000078237_100816_9PEZI1_v6.tar) |
| *Mycobacterium leprae* | Mycobacterium leprae | [UP000000806](https://www.uniprot.org/proteomes/UP000000806) | 1,602 | [177 MB](https://ftp.ebi.ac.uk/pub/databases/alphafold/latest/UP000000806_272631_MYCLE_v6.tar) |
| *Mycobacterium tuberculosis* | *M. tuberculosis* | [UP000001584](https://www.uniprot.org/proteomes/UP000001584) | 3,991 | [429 MB](https://ftp.ebi.ac.uk/pub/databases/alphafold/latest/UP000001584_83332_MYCTU_v6.tar) |
| *Neisseria gonorrhoeae* | *N. gonorrhoeae* | [UP000000535](https://www.uniprot.org/proteomes/UP000000535) | 2,106 | [195 MB](https://ftp.ebi.ac.uk/pub/databases/alphafold/latest/UP000000535_242231_NEIG1_v6.tar) |
| *Nocardia brasiliensis* | Nocardia brasiliensis | [UP000006304](https://www.uniprot.org/proteomes/UP000006304) | 8,398 | [873 MB](https://ftp.ebi.ac.uk/pub/databases/alphafold/latest/UP000006304_1133849_9NOCA1_v6.tar) |
| *Onchocerca volvulus* | Onchocerca volvulus | [UP000024404](https://www.uniprot.org/proteomes/UP000024404) | 12,039 | [1,621 MB](https://ftp.ebi.ac.uk/pub/databases/alphafold/latest/UP000024404_6282_ONCVO_v6.tar) |
| *Paracoccidioides lutzii* | Paracoccidioides lutzii | [UP000002059](https://www.uniprot.org/proteomes/UP000002059) | 8,794 | [1,294 MB](https://ftp.ebi.ac.uk/pub/databases/alphafold/latest/UP000002059_502779_PARBA_v6.tar) |
| *Plasmodium falciparum* | *P. falciparum* | [UP000001450](https://www.uniprot.org/proteomes/UP000001450) | 5,168 | [1,148 MB](https://ftp.ebi.ac.uk/pub/databases/alphafold/latest/UP000001450_36329_PLAF7_v6.tar) |
| *Pseudomonas aeruginosa* | *P. aeruginosa* | [UP000002438](https://www.uniprot.org/proteomes/UP000002438) | 5,555 | [613 MB](https://ftp.ebi.ac.uk/pub/databases/alphafold/latest/UP000002438_208964_PSEAE_v6.tar) |
| *Salmonella typhimurium* | *S. typhimurium* | [UP000001014](https://www.uniprot.org/proteomes/UP000001014) | 4,526 | [477 MB](https://ftp.ebi.ac.uk/pub/databases/alphafold/latest/UP000001014_99287_SALTY_v6.tar) |
| *Schistosoma mansoni* | Schistosoma mansoni | [UP000008854](https://www.uniprot.org/proteomes/UP000008854) | 9,735 | [1,802 MB)](https://ftp.ebi.ac.uk/pub/databases/alphafold/latest/UP000008854_6183_SCHMA_v6.tar) |
| *Shigella dysenteriae* | *S. dysenteriae* | [UP000002716](https://www.uniprot.org/proteomes/UP000002716) | 3,893 | [373 MB](https://ftp.ebi.ac.uk/pub/databases/alphafold/latest/UP000002716_300267_SHIDS_v6.tar) |
| *Sporothrix schenckii* | Sporothrix schenckii | [UP000018087](https://www.uniprot.org/proteomes/UP000018087) | 8,652 | [1,519 MB](https://ftp.ebi.ac.uk/pub/databases/alphafold/latest/UP000018087_1391915_SPOS1_v6.tar) |
| *Staphylococcus aureus* | *S. aureus* | [UP000008816](https://www.uniprot.org/proteomes/UP000008816) | 2,888 | [274 MB](https://ftp.ebi.ac.uk/pub/databases/alphafold/latest/UP000008816_93061_STAA8_v6.tar) |
| *Streptococcus pneumoniae* | *S. pneumoniae* | [UP000000586](https://www.uniprot.org/proteomes/UP000000586) | 2,031 | [202 MB](https://ftp.ebi.ac.uk/pub/databases/alphafold/latest/UP000000586_171101_STRR6_v6.tar) |
| *Strongyloides stercoralis* | Strongyloides stercoralis | [UP000035681](https://www.uniprot.org/proteomes/UP000035681) | 15,335 | [2,793 MB](https://ftp.ebi.ac.uk/pub/databases/alphafold/latest/UP000035681_6248_STRER_v6.tar) |
| *Trichuris trichiura* | Trichuris trichiura | [UP000030665](https://www.uniprot.org/proteomes/UP000030665) | 9,563 | [1,362 MB](https://ftp.ebi.ac.uk/pub/databases/alphafold/latest/UP000030665_36087_TRITR_v6.tar) |
| *Trypanosoma brucei* | Trypanosoma brucei | [UP000008524](https://www.uniprot.org/proteomes/UP000008524) | 8,491 | [1,345 MB](https://ftp.ebi.ac.uk/pub/databases/alphafold/latest/UP000008524_185431_TRYB2_v6.tar) |
| *Trypanosoma cruzi* | *T. cruzi* | [UP000002296](https://www.uniprot.org/proteomes/UP000002296) | 19,036 | [2,959 MB](https://ftp.ebi.ac.uk/pub/databases/alphafold/latest/UP000002296_353153_TRYCC_v6.tar) |
| *Wuchereria bancrofti* | Wuchereria bancrofti | [UP000270924](https://www.uniprot.org/proteomes/UP000270924) | 12,725 | [1,418 MB](https://ftp.ebi.ac.uk/pub/databases/alphafold/latest/UP000270924_6293_WUCBA_v6.tar) |
| *Arabidopsis thaliana* | *Arabidopsis* | [UP000006548](https://www.uniprot.org/proteomes/UP000006548) | 27,402 | [3,698 MB](https://ftp.ebi.ac.uk/pub/databases/alphafold/latest/UP000006548_3702_ARATH_v6.tar) |
| *Caenorhabditis elegans* | Nematode worm | [UP000001940](https://www.uniprot.org/proteomes/UP000001940) | 19,700 | [2,649 MB](https://ftp.ebi.ac.uk/pub/databases/alphafold/latest/UP000001940_6239_CAEEL_v6.tar) |
| *Candida albicans* | *C. albicans* | [UP000000559](https://www.uniprot.org/proteomes/UP000000559) | 5,973 | [981 MB](https://ftp.ebi.ac.uk/pub/databases/alphafold/latest/UP000000559_237561_CANAL_v6.tar) |
| *Danio rerio* | Zebrafish | [UP000000437](https://www.uniprot.org/proteomes/UP000000437) | 26,290 | [4,749 MB](https://ftp.ebi.ac.uk/pub/databases/alphafold/latest/UP000000437_7955_DANRE_v6.tar) |
| *Dictyostelium discoideum* | *Dictyostelium* | [UP000002195](https://www.uniprot.org/proteomes/UP000002195) | 12,612 | [2,187 MB](https://ftp.ebi.ac.uk/pub/databases/alphafold/latest/UP000002195_44689_DICDI_v6.tar) |
| *Drosophila melanogaster* | Fruit fly | [UP000000803](https://www.uniprot.org/proteomes/UP000000803) | 13,461 | [2,213 MB](https://ftp.ebi.ac.uk/pub/databases/alphafold/latest/UP000000803_7227_DROME_v6.tar) |
| *Escherichia coli* | *E. coli* | [UP000000625](https://www.uniprot.org/proteomes/UP000000625) | 4,370 | [456 MB](https://ftp.ebi.ac.uk/pub/databases/alphafold/latest/UP000000625_83333_ECOLI_v6.tar) |
| *Glycine max* | Soybean | [UP000008827](https://www.uniprot.org/proteomes/UP000008827) | 55,796 | [7,264 MB](https://ftp.ebi.ac.uk/pub/databases/alphafold/latest/UP000008827_3847_SOYBN_v6.tar) |
| *Homo sapiens* | Human | [UP000005640](https://www.uniprot.org/proteomes/UP000005640) | 23,586 | [4,938 MB](https://ftp.ebi.ac.uk/pub/databases/alphafold/latest/UP000005640_9606_HUMAN_v6.tar) |
| *Methanocaldococcus jannaschii* | *M. jannaschii* | [UP000000805](https://www.uniprot.org/proteomes/UP000000805) | 1,773 | [174 MB](https://ftp.ebi.ac.uk/pub/databases/alphafold/latest/UP000000805_243232_METJA_v6.tar) |
| *Mus musculus* | Mouse | [UP000000589](https://www.uniprot.org/proteomes/UP000000589) | 21,452 | [3,607 MB](https://ftp.ebi.ac.uk/pub/databases/alphafold/latest/UP000000589_10090_MOUSE_v6.tar) |
| *Oryza sativa* | Asian rice | [UP000059680](https://www.uniprot.org/proteomes/UP000059680) | 43,645 | [4,505 MB](https://ftp.ebi.ac.uk/pub/databases/alphafold/latest/UP000059680_39947_ORYSJ_v6.tar) |
| *Rattus norvegicus* | Rat | [UP000002494](https://www.uniprot.org/proteomes/UP000002494) | 22,152 | [3,602 MB](https://ftp.ebi.ac.uk/pub/databases/alphafold/latest/UP000002494_10116_RAT_v6.tar) |
| *Saccharomyces cerevisiae* | Budding yeast | [UP000002311](https://www.uniprot.org/proteomes/UP000002311) | 6,055 | [977 MB](https://ftp.ebi.ac.uk/pub/databases/alphafold/latest/UP000002311_559292_YEAST_v6.tar) |
| *Schizosaccharomyces pombe* | Fission yeast | [UP000002485](https://www.uniprot.org/proteomes/UP000002485) | 5,196 | [803 MB](https://ftp.ebi.ac.uk/pub/databases/alphafold/latest/UP000002485_284812_SCHPO_v6.tar) |
| *Zea mays* | Maize | [UP000007305](https://www.uniprot.org/proteomes/UP000007305) | 39,139 | [4,792 MB](https://ftp.ebi.ac.uk/pub/databases/alphafold/latest/UP000007305_4577_MAIZE_v6.tar) |

Note: *Enterococcus faecium* ([UP000325664](https://www.uniprot.org/proteomes/UP000325664)) and *Mycobacterium ulcerans* ([UP000020681](https://www.uniprot.org/proteomes/UP000020681)) are not included in v6 of the database.

**The Encyclopedia of Domains (TED) Q-score**

The Qscore is a weighted average of structural alignment coverage (0.5 weight), TED consensus level (0, 50 or 100 for low, medium and high respectively, 0.125 weight), domain compactness (globularity)(0 or 100 for loose or compact respectively, 0.125 weight), normalised mean pLDDT score (0.125 weight) and a modified e-value factor ( 0.125 weight). Based on the Qscore and CATH assignment, each domain is given a quality label: “High-confidence” (Qscore ≥ 75 and full 4-level C.A.T.H assignment), “Moderate”(Qscore ≥ 70 and 3-or 4-level C.A.T/C.A.T.H assignment) or “Uncertain” (lack of a 3- or 4-level assignment, regardless of the Qscore).

As several components reflect structural order and AlphaFold model confidence, predicted domains with shallow MSAs, limited templates, or flexible packing can obtain lower Qscores. Such reductions indicate uncertainty in the predicted 3D arrangement rather than implying that the region is intrinsically disordered. Insertions that are not part of the core structural unit are also ignored by TED, often annotated as discontinuous segments, and do not contribute to the domain’s Qscore. While users should interpret low Qscores with caution, it signals for structural reliability of the domain model, and not as evidence against function or conservation of the underlying sequence. This has been illustrated with numerous examples that show similar sequences with high Qscores compared with low-confidence/poorly folded predictions (low Qscore) in supplementary figure 16 of Lau et al. (2024) (1).

**References**

[1. Lau,A.M., Bordin,N., Kandathil,S.M., Sillitoe,I., Waman,V.P., Wells,J., Orengo,C.A. and Jones,D.T. (2024) Exploring structural diversity across the protein universe with The Encyclopedia of Domains. *Science*, **386**, eadq4946.](https://www.zotero.org/google-docs/?XGXWLr)
